# Effects of sprayer speed, spray distance, and nozzle arrangement angle on low-flow air-assisted spray deposition

**DOI:** 10.3389/fpls.2023.1184244

**Published:** 2023-05-08

**Authors:** Shiqun Dai, Mingxiong Ou, Wentao Du, Xuejun Yang, Xiang Dong, Li Jiang, Tie Zhang, Suming Ding, Weidong Jia

**Affiliations:** ^1^ School of Agricultural Engineering, Jiangsu University, Zhenjiang, China; ^2^ Science Innovation Center, Chinese Academy of Agriculture Mechanization Sciences Group Co., Ltd., Beijing, China; ^3^ Nanjing Institute of Agricultural Mechanization, Ministry of Agriculture and Rural Affairs, Nanjing, China

**Keywords:** air-assisted, sprayer speed, spray distance, nozzle arrangement angle, deposit coverage, spray penetration, deposit distribution

## Abstract

Air-assisted spraying technology is widely used in orchard sprayers to disturb canopy leaves and force droplets into the plant canopy to reduce droplet drift and increase spray penetration. A low-flow air-assisted sprayer was developed based on a self-designed air-assisted nozzle. The effects of the sprayer speed, spray distance, and nozzle arrangement angle on the deposit coverage, spray penetration, and deposit distribution were investigated in a vineyard by means of orthogonal tests. The optimal working conditions for the low-flow air-assisted sprayer working in the vineyard were determined as a sprayer speed of 0.65m/s, a spray distance of 0.9m, and a nozzle arrangement angle of 20°. The deposit coverages of the proximal canopy and intermediate canopy were 23.67% and 14.52%, respectively. The spray penetration was 0.3574. The variation coefficients of the deposit coverage of the proximal canopy and intermediate canopy, which indicate the uniformity of the deposition distribution, were 8.56% and 12.33%, respectively.

## 1 Introduction

Although great progress has been made in the development of plant protection machinery, pests and diseases continue to affect vineyards (Wise et al., 2010). The most effective and economical method of crop protection for pest and disease control in orchards remains the chemical control method (Gong et al., 2020). Pesticides are widely used in vineyards, but excessive pesticide spraying can lead to human damage and environmental pollution (Abd Kharim et al., 2019; Sinha et al., 2020). At the same time, the dense grape canopy reduces the effectiveness of pesticides to control pests and diseases inside the plant (Simone et al., 2016). Therefore, vineyard sprayers have been selected for air-assisted droplet transport to and penetration of the canopy (Miranda-Fuentes et al., 2018).

Since the 1950s, axial fan airblast sprayers have been used in orchard plant protection (Fox et al., 2008). Axial fan airblast sprayers are widely used because of their strong auxiliary airflow and long range. With the change in the modern orchard planting pattern, for use in typical dwarf and semi-dwarf orchards, droplets of axial fan airblast sprayers are easily dispersed in the air, causing environmental pollution problems (Blanco et al., 2019). As a result, tower sprayers are beginning to be used in modern orchards. Studies have proven that droplet drift is more severe in axial fan sprayers compared to tower sprayers (Landers et al., 2017). Different types of sprayers have been developed in order to improve the effectiveness of the sprayers, such as multi-airway sprayers (Grella et al., 2020), multi-row sprayers (Pergher and Zucchiatti, 2018), or individual outlet sprayers (Miranda-Fuentes et al., 2018). To reduce pesticide use and improve pesticide deposition on grape leaves, an electrostatic sprayer using an innovative pneumatic electrostatic sprayer was developed (Simone and Emanuele, 2015).

During the operation of the orchard sprayer, the working parameters have a large influence on the deposition of droplets in the canopy (Sinha et al., 2020). Different liquid flow rates, air flow rates, forward speed, targeted and wind-oriented airflow adjustment, target height, and orientation can all affect the effectiveness of the sprayer (Devanand and Divaker, 2010; Ryszard et al., 2017; Salcedo et al., 2020). Choosing the right working conditions for the sprayer can effectively improve the quality of the sprayer’s operation.

In this paper, a low-flow air-assisted sprayer for vineyards was designed based on a self-designed air-assisted nozzle to reduce the amount of pesticide spraying (Dai et al., 2022). In order to improve the quality of sprayer operation, an orthogonal test on three factors, namely, sprayer speed, spray distance, and the nozzle arrangement angle, was designed to determine the optimal working conditions of the sprayer. The data were also analyzed for the deposit coverage, spray penetration, and deposit distribution after the orthogonal tests, and the influence pattern between the three factors and each index was studied. We hope that the research results will provide some assistance in future air-assisted sprayer operations in vineyards.

## 2 Materials and methods

### 2.1 Sprayer characteristics

**Figure 1:**
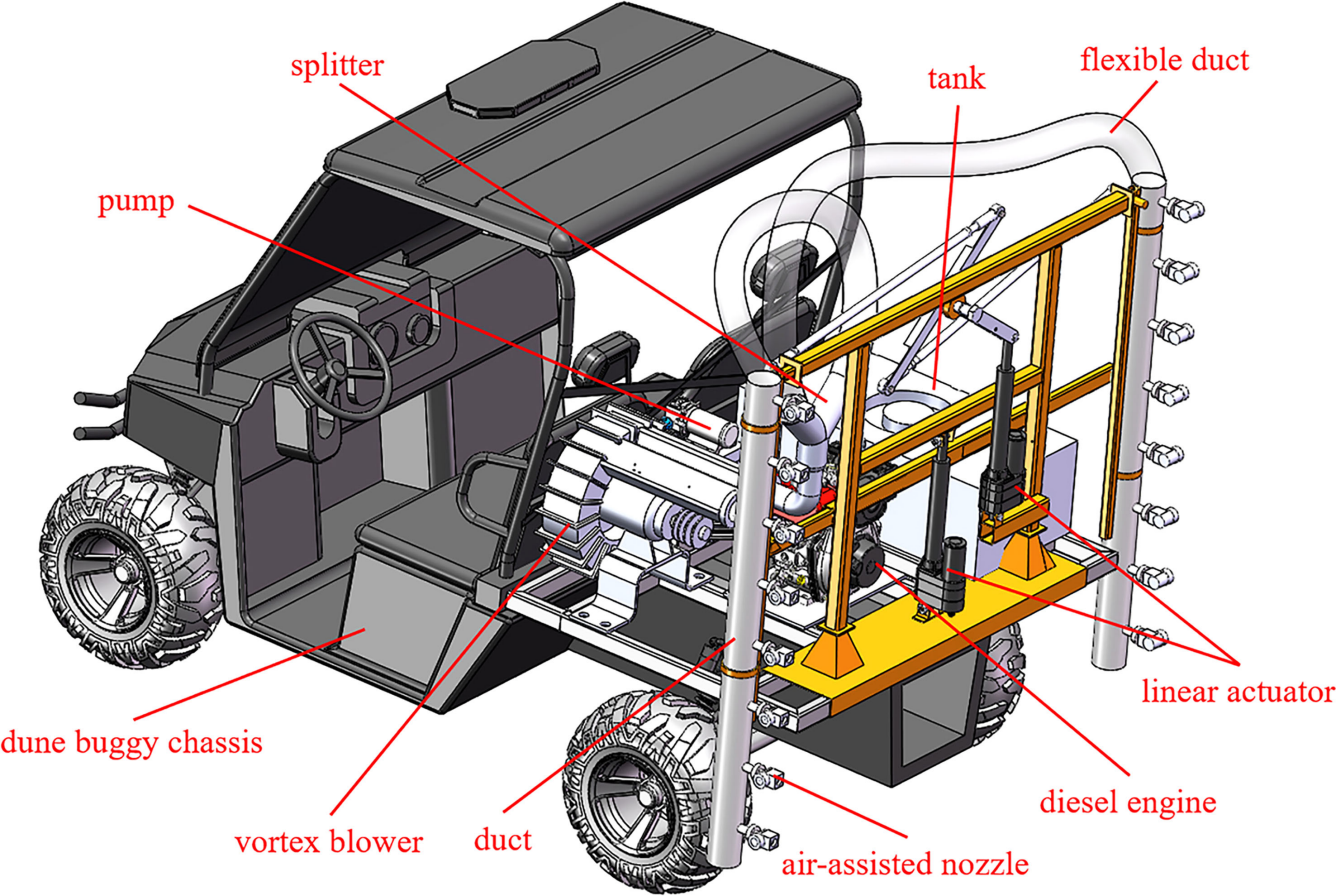
The low-flow air-assisted sprayer structure schematic.

A low-flow air-assisted sprayer was developed as shown in **Figure 1**. The length, width, and height of the sprayer are 2.8m, 1.5m, and 2m, respectively. This low-flow air-assisted sprayer mainly consists of a travel system, an air-assisted system, and a spraying system. To be suitable for vineyards with soft soil, the travel system of the sprayer adopts 4WD dune buggy chassis by the China Jiangsu LINHAI Group, of which the Chassis power is 14.1kW. The air-assisted system mainly includes a Vortex blower to provide high-pressure airflow, a diesel engine to power the Vortex blower, ducts to fix the air-assisted nozzle, a splitter to divide the airflow evenly to both sides of the duct, and flexible ducts to guide the flow. The power rating of the Vortex blower (YASHIBA HG810-75CS9, China) is 7.5kW and the diesel engine power (BEILONG 2V95, China) is 9kW. The spraying system mainly consists of an electric water pump powered by a 12V battery, a 200L polyethylene tank, and air-assisted nozzles. The air-assisted nozzle is a self-designed low-flow nozzle (Dai et al., 2022). When the air pressure is 0.5bar and the liquid pressure is 0.7bar, the spray angle of the air-assisted nozzle is 18°, the volume flow rate is 0.21L/min, and the volume median diameter of the droplet is 35µm. There are 2 rows of air-assisted nozzles on both sides of the sprayer and there are 8 air-assisted nozzles in each row. The air-assisted nozzles can be arranged tilted downwards at an angle perpendicular to the duct. This angle was named the nozzle arrangement angle (Ferguson et al., 2016). Two linear actuators were used to facilitate the adjustment of the height of the nozzle and the distance from the nozzle to the vine canopy.

### 2.2 Field test site and canopy characterization

Field tests were conducted to study the effect of the low-flow air-assisted sprayer and to determine the optimal operating conditions in the field. Field tests were performed on 11-12th August 2021 in a research vineyard located in Yuquanying Farm, Ningxia Hui Autonomous Region, China (38.13° N, 105.96° E). During the tests, the weather was breezy without rain. The temperature in the field during the daytime was 20 to 24°C and the relative humidity was 46 ± 3%. Ten measurements of wind speed were taken using an anemometer (testo 416, Germany) at five-minute intervals. The maximum wind speed was 2.2m/s and the average wind speed was 1.3m/s.

**Figure 2:**
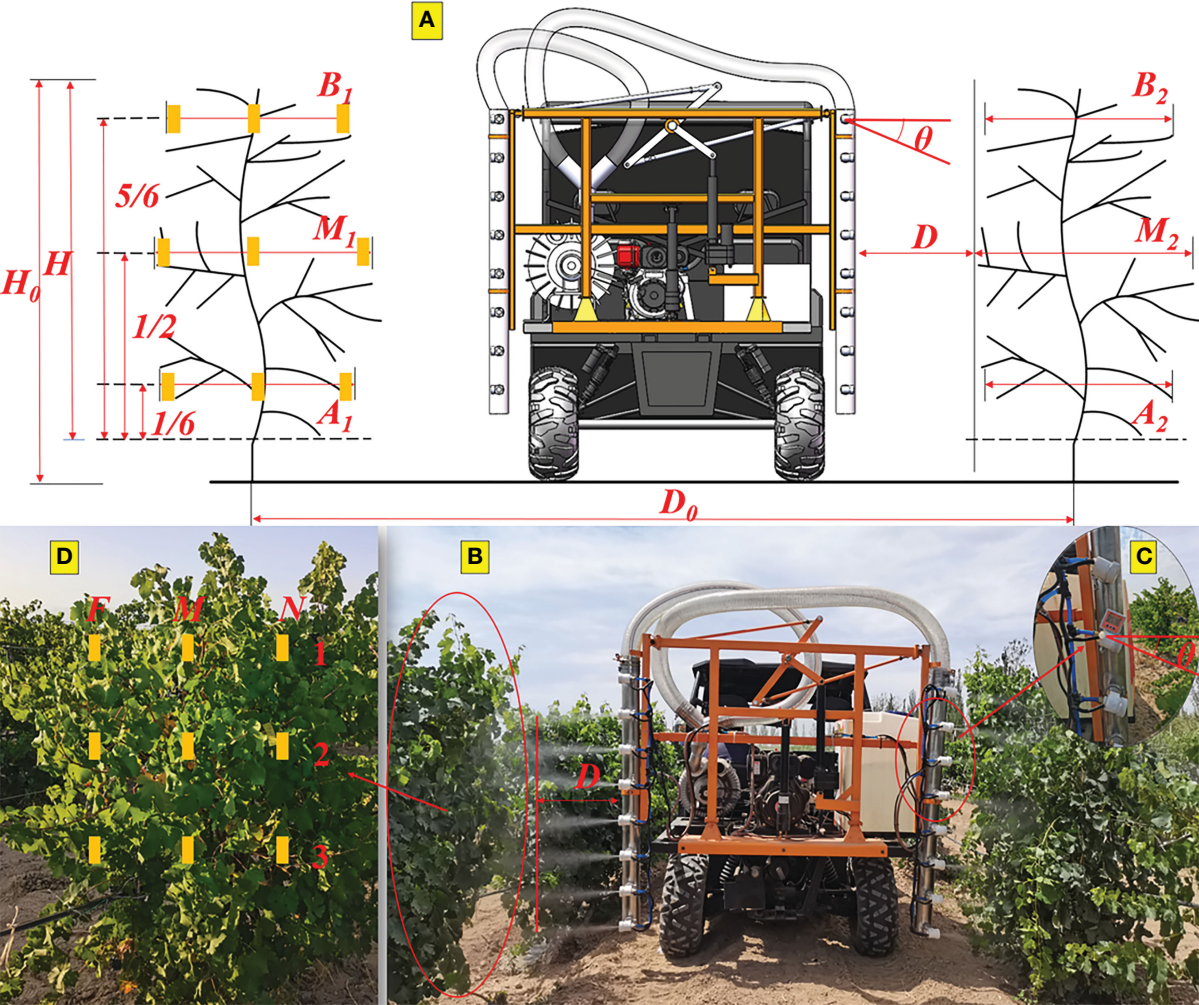
**(A)** Diagram of grape canopy and spraying operation; **(B)** graph of the sprayer in real time; **(C)** diagram of nozzle arrangement angle; **(D)** diagram of water-sensitive paper arrangement. Yellow marks represent water-sensitive papers.


The Merlot vines trained using the spur cordon system and the row spacing (*D_0_*) of the research vineyard were about 3.5m (Otto et al., 2013). The height of the vine (*H_0_*) and canopy (*H*) was about 2m and 1.8m, respectively, and the thickness of the canopy was about 0.8 to 1.2m. During the spray test phase, grapes were in the full leaf stage, and the leaf area index (*LAI*) was about 3.63. The model of the plant row volume (*PRV*) was used to calculate the *LAI* of the grape canopy (Sanchez-Hermosilla et al., 2013). Because the grape canopy thickness varied at each height, the canopy thickness at 1/6, 1/2, and 5/6 was taken to estimate the *PRV* in **Figure 2A**. The *LAI* and *PRV* were calculated as shown below.

(1) $$LAI=PRV^{1.25}\times 3.5\times 10^{-5}$$

(2) $$PRV=\frac{10000H\left[\left(A_{1}+A_{2})+(M_{1}+M_{2})+(B_{1}+B_{2}\right)\right]}{3D_{0}}$$

where *H* is the grape canopy height (m), *A_1_* and *A_2_* are the thickness of the canopy at 1/6 the height (m), *B_1_* and *B_2_* are the thickness of the canopy at 5/6 the height (m), *M_1_* and *M_2_* are the thickness of the canopy at 1/2 the height (m), and *D* is the distance between the rows (m).

### 2.3 Field test design

Regarding the sprayer’s spraying operations, the deposit coverage on the leaves was significantly influenced by the sprayer speed (*v*), the nozzle arrangement angle (*θ*) and the spray distance (*D*) (Devanand and Divaker, 2010). The sprayer speed (*v*) is the sprayer’s forward speed and three levels of sprayer speed (0.65m/s, 0.9m/s and 1.15m/s) are chosen referring to the speed of orchard sprayer (Simone et al., 2016). The spray distance (*D*) is the distance between the nozzles and grape canopy as shown in **Figure 2B**, and the appropriate spray distance levels (0.6m, 0.9m and 1.2m) are selected based on the wind attenuation performance of the air-assisted nozzle. The nozzle arrangement angle (*θ*) is the angle at which the spray nozzles are arranged tilted downwards at an angle perpendicular to the duct as shown in **Figure 2C**. Too large nozzle arrangement angle would increase the spray distance, however, too small nozzle arrangement angle will be due to errors and other reasons to make the results insignificant. Therefore, the choice of nozzle arrangement angle of 0°, 10° and 20°, respectively. To explore the effect of the sprayer speed, nozzle arrangement angle, and spray distance on the deposit coverage of spraying operations and find the optimal combination, orthogonal tests with three factors, each at three levels (**Table 1**), were designed and carried out, which generated nine different component ratios, and the details are shown in **Table 2**.

**Table 1:** Factors and levels of the orthogonal experiment.

Treatment no.	A sprayer speed v (m/s)	B spray distance D (m)	C nozzle arrangement angle θ (°)
*Level 1*	0.65	0.6	0
*Level 2*	0.9	0.9	10
*Level 3*	1.15	1.2	20

**Table 2:** Schemes of the orthogonal experiment.

Treatment no.	A	B	Empty column	C
1	1	1	1	1
2	1	2	2	2
3	1	3	3	3
4	2	1	2	3
5	2	2	3	1
6	2	3	1	2
7	3	1	3	2
8	3	2	1	3
9	3	3	2	1

To prevent an inaccurate nozzle arrangement angle due to uneven ground in the test area, the sprayer passed through the test area several times to compact the ground into a specific track. The sprayer followed a specific track for spraying operations, and the distance between the nozzles and the grape canopy was controlled by adjusting the linear actuator. As the sprayer passed through the test area, the sprayer speed was controlled by feedback from the dashboard speed display to ensure that the speed was near the target speed.

During the sprayer spraying operation, the air pressure was 0.5 bar, the liquid pressure was 0.7 bar, and the volume flow rate was 3.36 L/min. The errors of the sprayer speed, spray distance, and nozzle arrangement angle were within ±0.05 m/s, ± 0.08 m, and ±5°, respectively.

Water-sensitive paper (26×76mm, Teejet, USA) was used to capture droplets sprayed by sprayers in these tests. According to the height of the grape canopy, water-sensitive papers were arranged in three layers, at 5/6, 1/2, and 1/6 height of the grape canopy, named layer 1, 2, and 3, respectively. According to the thickness of the grape canopy, water-sensitive papers were also arranged in three layers, at both sides and at the center of the grape canopy, named layer N, M, and F from near to far from the sprayer, respectively. For example, the water-sensitive paper, which was arranged at 1/2 height and on the far side of the grape canopy, was marked as F2. Nine sites per test were arranged, as shown in **Figure 2D**. After the test, the water-sensitive paper was left to dry completely, then marked and bagged for post-processing. Each test was repeated three times. The sprayer sprayed water in each test.

### 2.4 Field tests data analyses

In field trials, water-sensitive paper is widely used to assess the deposit coverage of droplets on leaves (Kosuke et al., 2012; Ömer and Ali, 2020). So, the leaves deposit coverage was studied in this paper (Victor et al., 2020). All water-sensitive papers were scanned by a scanner (M7628DNA, LENOVO) at 600 dpi to obtain 8-bit grayscale images. A software entitled “DepositScan”, which was developed by the USDA-ARS Application Technology Research Unit, was used to obtain the leaves deposit coverage (Zhu et al., 2011).

In this paper, *C_ij_* represented the deposit coverage of layer *ij* (*i*=N, M and F; *j*=1, 2 and 3). *C_i_* included *C_i1_*, *C_i2_*, and *C_i3_*. Because each test was repeated three times, *C_i_* contained nine samples. In the data analysis, the two maximum and two minimum samples values were removed and the average of the remaining five samples was used as *C_i_*. The deposit coverage of the whole grape canopy (*C_W_*, %) was calculated as follows:


(3) $$C_{W}=C_{N}+C_{M}+C_{F}$$

The spray penetration (*SP*) in the canopy was calculated as the ratio of the layer M deposit coverage (*C_M_*, %) and the whole grape canopy deposit coverage (*C_W_*, %) (Li et al., 2022):


(4) $$SP=\frac{C_{M}}{C_{W}}$$

The deposit distribution is also an important indicator to evaluate the effectiveness of the sprayer operation, which can be described by the variation coefficient of the deposit coverage in the same canopy. The variation coefficient of the deposit coverage in layer I (*CV_I_*) was studied as shown in Equation 5 (Liu et al., 2021):


(5) $$CV_{I}=\frac{\sqrt{\sum_{j=1}^{n}{\left(C_{I\text{j}}^{`}-\bar{C_{I}}\right)}^{2}/(n-1)}}{\bar{C_{I}}}\times 100\%$$

where *I* represents *M* and *N;C^`^_Ij_* represents the average of *C_Ij_* in three replicate experiments, %; $\bar{C_{I}}$ is the average of nine *C_I_* samples, %; and *n* is the number of layers of the vertically oriented water-sensitive paper sites.

## 3 Results and discussion

### 3.1 Deposit coverage

The deposit coverage of the proximal canopy (*C_N_*) and the deposit coverage of the intermediate canopy (*C_M_*) were studied in **Table 3** by processing data from orthogonal trials in the field. A higher deposit coverage indicates better results from the sprayer.

**Table 3:** Deposit coverage of the field orthogonal test.

Treatment no.	A	B	Empty column	C	C_N_ (%)	C_M_ (%)
1	1	1	1	1	24.86	14.61
2	1	2	2	2	22.22	13.33
3	1	3	3	3	14.39	6.35
4	2	1	2	3	22.00	11.98
5	2	2	3	1	20.53	7.42
6	2	3	1	2	8.26	3.78
7	3	1	3	2	13.24	7.72
8	3	2	1	3	14.35	7.44
9	3	3	2	1	7.70	2.70
*K1(C_N_)*	61.47	60.10	47.46	53.09		
*K2(C_N_)*	50.78	57.09	51.91	43.72		
*K3(C_N_)*	35.29	30.35	48.16	50.74		
*k1(C_N_)*	20.49	20.03	15.82	17.70		
*k2(C_N_)*	16.93	19.03	17.30	14.57		
*k3(C_N_)*	11.76	10.12	16.05	16.91		
*R(C_N_)*	8.73	9.92	1.48	3.12		
*K1(C_M_)*	34.29	34.32	25.84	24.73		
*K2(C_M_)*	23.18	28.19	28.01	24.84		
*K3(C_M_)*	17.87	12.83	21.49	25.78		
*k1(C_M_)*	11.43	11.44	8.61	8.24		
*k2(C_M_)*	7.73	9.40	9.34	8.28		
*k3(C_M_)*	5.96	4.28	7.16	8.59		
*R(C_M_)*	5.47	7.16	2.17	0.35		

As shown in **Table 3**, for *C_N_*, the maximum was 24.86% and the minimum was 7.70% among the nine sets of experiments in the orthogonal test, with a large difference. However, for *C_M_*, the maximum value was 14.61% and the minimum was 2.70%. *C_M_* is about half of *C_N_*, or even smaller. There are two main reasons for this. First, droplets can be lost during transportation due to the drifting of the droplets caused by external factors (Grella et al., 2020; Rathnayake et al., 2021). Layer N was closer to the sprayer than layer M, and more droplets were adsorbed on the water-sensitive paper of layer N. Second, the water-sensitive papers of layer N were arranged on the surface of the canopy, and there was not much canopy obstruction between them and the sprayer; the water-sensitive paper of layer M was located in the center of the canopy, and the canopy leaves acted as an obstruction to the droplets (Duga et al., 2015), so fewer droplets penetrated the center of the canopy and were adsorbed on the water-sensitive paper of M.

**Figure 3:**
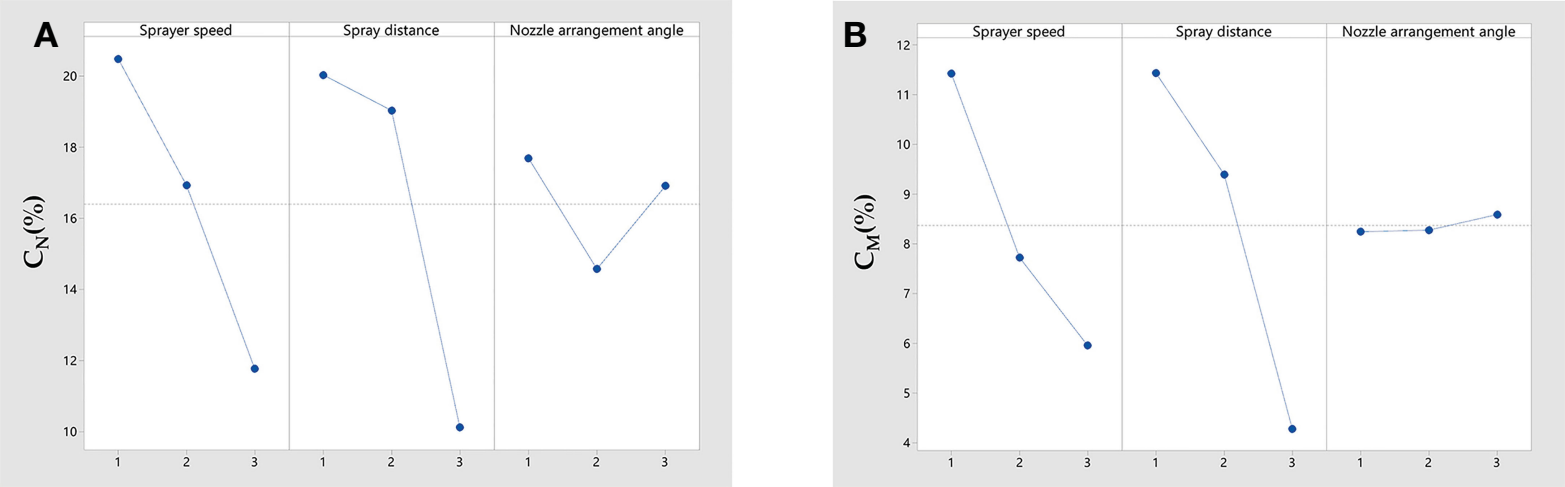
Range analysis of *C_N_* **(A)** and *C_M_* **(B)**.

For *C_N_* and *C_M_*, RB>RA>RC; this means that the indicator which had the largest impact was the spray distance, followed by the sprayer speed, and the indicator with the smallest impact was the nozzle arrangement angle. Based on the data from the range analysis in **Table 3**, the relationship between the effects of the three factors on *C_N_* and *C_M_* was plotted as shown in **Figure 3**. As shown in **Figure 3**, the sprayer speed and spray distance showed the same trend, with both *C_N_* and *C_M_* showing a decreasing trend as the sprayer speed and spray distance increased; Salyani obtained similar results in a wind-delivered spray test studying citrus trees (Salyani, 2000). Increasing the sprayer speed can produce airflow in opposite directions, leading to changes in the auxiliary airflow angles, which may also lead to droplet drift loss in complex airflow fields and affect the droplet deposition (Triloff, 2018). The nozzle arrangement angle had an insignificant pattern of influence on *C_N_* and had a catalytic effect, but the impact was not significant on *C_M_*. Foque’s study of laurel spraying operations yielded similar conclusions (Foqué et al., 2012).

The optimal combination of *C_N_*, which was determined according to the average values of the three factors at different levels, is indicated by B1A1C1, but there were differences in the optimal combination of *C_M_*, which is indicated by B1A1C3.

### 3.2 Spray penetration

Spray penetration (*SP*) can effectively reflect the droplet’s deposition in the canopy. The results of the field orthogonal test were processed according to the calculation formula of the spray penetration, as shown in the **Table 4**. The larger the value of the spray penetration, the better the droplet deposition in the canopy.

**Table 4:** Spray penetration of the field orthogonal test.

Treatment no.	A	B	Empty column	C	SP
1	1	1	1	1	0.3388
2	1	2	2	2	0.3488
3	1	3	3	3	0.3340
4	2	1	2	3	0.3256
5	2	2	3	1	0.2581
6	2	3	1	2	0.2845
7	3	1	3	2	0.3177
8	3	2	1	3	0.3112
9	3	3	2	1	0.2348
K1	1.0215	0.9820	0.9345	0.8316	
K2	0.8682	0.9180	0.9092	0.9510	
K3	0.8636	0.8533	0.9097	0.9707	
k1	0.3405	0.3273	0.3115	0.2772	
k2	0.2894	0.3060	0.3031	0.3170	
k3	0.2879	0.2844	0.3032	0.3236	
R	0.0526	0.0429	0.0083	0.0464	

**Figure 4:**
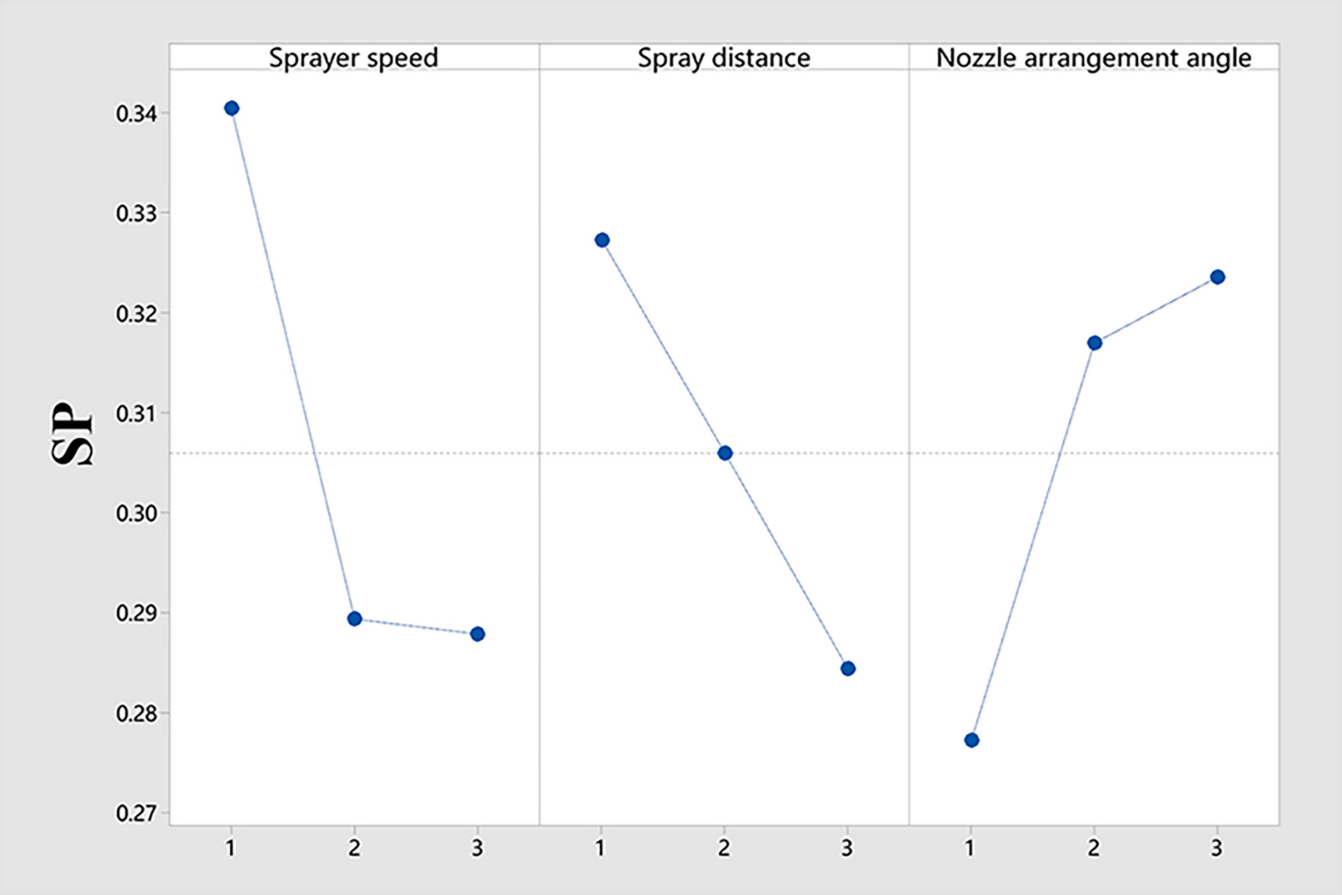
Range analysis of spray penetration.

As shown in **Table 4**, the spray penetration was basically around 0.2~0.3, without much difference. This proves that the low-flow air-assisted sprayer operates with a better spray penetration. RA>RC>RB means that the sprayer speed had the greatest impact on the spray penetration, followed by the nozzle arrangement angle, and finally the spray distance, which had relatively little effect. Based on the data in **Table 4**, **Figure 4** is drawn to show the relationship between the influence of each factor on the spray penetration. From **Figure 4**, it can be seen that the sprayer speed, spray distance, and nozzle arrangement angle had a clear pattern on the spray penetration. The difference is that the spray penetration decreased with the increase in the sprayer speed and spray distance, while the spray penetration increased with the increase in the nozzle arrangement angle.

The auxiliary airflow disturbed the canopy foliage and helped the droplets to enter the interior of the canopy, which was the main factor affecting the droplet penetration (Triloff, 2018; Bernat et al., 2022). An increase in the sprayer speed led to a decrease in the wind volume per unit canopy area, which was insufficient to disturb the blades to allow the droplets to enter the interior of the canopy, resulting in reduced penetration. After the auxiliary airflow was emitted from the nozzle, the airflow speed decreased rapidly against the increase in the spray distance (Gu et al., 2014). The increase in the spray distance led to a decrease in the velocity of the auxiliary airflow, which was insufficient to sufficiently disturb the canopy foliage, reducing penetration.

The nozzle arrangement angle had a significant impact on the spray penetration; within the test nozzle installation angle, the greater the angle, the better the spray penetration. Similar findings have been reported in previous studies (de Lima et al., 2018). The nozzle arrangement angle is the angle between the nozzle and the grape canopy. Therefore, changing the nozzle mounting angle and thus the angle of contact between the auxiliary airflow and the canopy may make it easier for the auxiliary airflow to disturb the canopy blades and facilitate the entry of droplets into the interior of the canopy. This is only a conjecture, and the exact reason for this requires further investigation. However, in the field of UAV research, the same principle may exist in the experimental study of pear orchards by changing the angle of the wing while changing the angle of the nozzle, which effectively improves the coverage inside the canopy (Qi et al., 2022).

The optimal combination determined according to the analysis of the influencing reasons of the three factors is A1C3B1.

### 3.3 Deposit distribution

The variation coefficients of the deposit coverage in layer M (*CV_M_*) and in layer N(*CV_N_*), which indicate the uniformity of the deposition distribution, are important parameters for evaluating the sprayer operation, and the same can be said for the spray penetration. The data of *CV_M_* and *CV_N_* from the field orthogonal trials are shown in **Table 5**. Smaller data means a better sprayer operation.

**Table 5:** *CV_N_* and *CV_M_* of the field orthogonal test.

Treatment no.	A	B	Empty column	C	CV_N_ (%)	CV_M_ (%)
1	1	1	1	1	15.23	30.56
2	1	2	2	2	11.76	16.15
3	1	3	3	3	6.24	33.36
4	2	1	2	3	19.58	29.06
5	2	2	3	1	18.84	28.21
6	2	3	1	2	10.18	33.08
7	3	1	3	2	25.15	20.59
8	3	2	1	3	16.10	19.77
9	3	3	2	1	23.69	44.59
*K1(CV_N_)*	33.23	59.97	41.52	57.76		
*K2(CV_N_)*	48.61	46.70	55.03	47.09		
*K3(CV_N_)*	64.95	40.11	50.23	41.92		
*k1(CV_N_)*	11.08	19.99	13.84	19.25		
*k2(CV_N_)*	16.20	15.57	18.34	15.70		
*k3(CV_N_)*	21.65	13.37	16.74	13.97		
*R(CV_N_)*	10.57	6.62	4.50	5.28		
*K1(CV_M_)*	80.07	80.21	83.40	103.36		
*K2(CV_M_)*	90.36	64.13	89.81	69.82		
*K3(CV_M_)*	84.94	111.03	82.16	82.19		
*k1(CV_M_)*	26.69	26.74	27.80	34.45		
*k2(CV_M_)*	30.12	21.38	29.94	23.27		
*k3(CV_M_)*	28.31	37.01	27.39	27.40		
*R(CV_M_)*	3.43	15.63	2.55	11.18		

**Figure 5:**
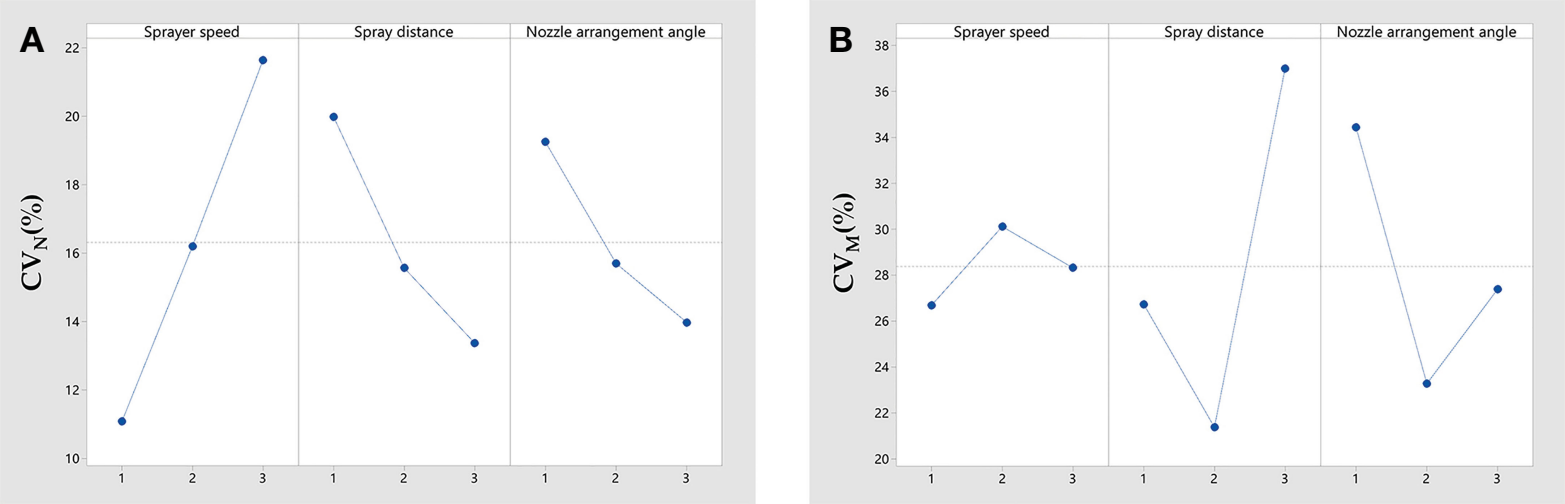
Range analysis of *CV_N_* **(A)** and *CV_M_* **(B)**.

As shown in **Table 5**, it can be seen that RA>RB>RC, the three factors which influence *CV_N_* in order of importance, are the sprayer speed, spray distance, and nozzle arrangement angle. According to the data in **Table 5**, the patterns of influence of each factor on *CV_N_* is further elucidated in **Figure 5A**. The pattern of influence of the three factors on *CV_N_* is more obvious. The effect of the sprayer speed on *CV_N_* is positively correlated. High sprayer speeds can lead to a poor deposition distribution, resulting in an elevated *CV_N_*, and this has been confirmed in previous studies (Planas et al., 1998; Devanand and Divaker, 2010). As mentioned earlier, a higher sprayer speed affects the droplet deposition; there is more uncertainty regarding this effect, which further affects the deposition uniformity. The effect of the spray distance on *CV_N_* is negatively correlated. Contrary to the speed of the sprayer, a better deposition distribution was obtained for longer spray distances. This may be due to the fact that although longer spray distances lead to increased droplet drift, the inertial force of the droplets overcomes the external forces causing a drift at the spray distances tested, thus improving the droplet deposition uniformity. However, this conjecture needs to be corroborated by further experimental studies. Similarly, the increase in the nozzle installation angle also improved the uniformity of the mist distribution. Foque’s experimental study of spraying on laurel trees improved the deposition distribution significantly by changing the spray direction (Foqué et al., 2012). Although the angle of change varies, it was confirmed that the angle has an effect on the uniformity of the deposition distribution.

Noted in **Table 5**, we found that the variation coefficient of the deposit coverage in layer N(*CV_N_*) reached a maximum of 25.15% and a minimum of 6.24%, while the variation coefficient of the deposit coverage and in layer M(*CV_M_*) reached a maximum of 44.59% and a minimum of 16.15%. Comparing the magnitude of the *CV_N_* and *CV_M_* values, *CV_M_* was generally significantly larger than *CV_N_*, indicating a difference of about twice. At the same time, regarding M, RB>RC>RA, which means that the spray distance had the greatest influence on *CV_M_*, followed by the nozzle arrangement angle, and the sprayer speed had the least influence. The order of the magnitude of the effects of the three factors on the *CV_M_* is different from that of the *CV_N_*. Additionally, then, compared with the pattern of *CV_N_*, the pattern of the influence of each factor on *CV_M_* appears to be more chaotic and there is no regularity, as demonstrated in **Figure 5B**. Compared to *CV_N_*, the above three differences exist in the study of *CV_M_*. The main reason for these would be that the sampling site N is located at the edge of the grape canopy and sampling site M is located in the middle of the grape canopy. Uncertainties such as the leaf density of the canopy, porosity, and the growth direction of the leaves, which exist stochastically, greatly influence the attachment of the droplets (Duga et al., 2015).

The optimal combination of *CV_N_*, which is determined according to the average values of the three factors at different levels, is A1B3C3, but the optimal combination of *CV_M_* is B2C2A1.

### 3.4 Optimal working condition

During the data analysis of the orthogonal test, different indicators correspond to different optimal working conditions. An optimal working condition needs to be determined in the actual operation. A slower sprayer speed can obtain a higher deposit coverage and spray penetration, as well as a better deposition distribution uniformity, so a slower sprayer speed is the appropriate choice. The angle of the nozzle arrangement has a greater effect on the spray penetration and deposit distribution, and a smaller effect on the deposition coverage. A larger nozzle arrangement angle can increase the spray penetration and improve the deposit distribution uniformity, so a larger nozzle arrangement angle is preferred. The spray distance contributes to the deposit coverage and spray penetration, but it reduces the deposit distribution uniformity, indicating a paradoxical combination. However, a suitable spray distance can reduce the uniformity of fog distribution in the middle of the canopy. So, we compromised by selecting the middle spray distance.

In summary, A1B2C3 was used as a low-flow air-assisted sprayer for field work conditions. A field test was conducted with this working condition to obtain the deposit coverage, spray penetration, and deposit distribution uniformity, as shown in **Table 6**.

**Table 6:** Optimal working condition field test results.

Type	C_N_ (%)	C_M_ (%)	SP	CV_N_ (%)	CV_M_ (%)
A1B2C3	23.67	14.52	0.3574	8.56	12.33

## 4 Conclusion

A low-flow air-assisted sprayer was designed for use in a vineyard, and field trials were conducted using an orthogonal test with the sprayer speed, spray distance, and nozzle arrangement angle. The deposit coverage, spray penetration, and deposit distribution were studied in the orthogonal test.

The spray distance had the largest influence on the deposit coverage, followed by the sprayer speed, and finally, the nozzle arrangement angle had the smallest influence. The spray distance and sprayer speed had a large and negative effect on the deposit coverage, but the angle of the nozzle arrangement had a smaller effect on the deposit coverage. The order of influence on the spray penetration was sprayer speed, the nozzle mounting angle, and spray distance. The increase in the sprayer speed and spray distance reduced the spray penetration, but the nozzle arrangement angle promoted spray penetration. The effect pattern of the sprayer speed, spray distance, and nozzle arrangement angle on the deposit distribution of canopy layer N was obvious. The sprayer speed had the greatest effect, and a higher sprayer speed reduced the deposit distribution uniformity. The spray distance and nozzle arrangement angle had relatively small effects, but both improved the deposit distribution uniformity. For the middle canopy layer M, uncertainties such as the leaf density of the canopy, porosity, and the growth direction of the leaves, which exist stochastically, greatly influenced the attachment of the droplets. Therefore, there was no significant trend of the three factors on the deposition uniformity.

The optimal working condition of the low-flow air-assisted sprayer was determined to be sprayer speed 0.65m/s, spray distance 0.9m, and nozzle arrangement angle 20° by considering the effects of the sprayer speed, spraying distance, and nozzle arrangement angle on each index, and a field test was conducted. The deposit coverages of the proximal canopy (*C_N_*) and intermediate canopy (*C_M_*) were 23.67% and 14.52%, respectively. The spray penetration was 0.3574. The variation coefficients of the deposit coverage of the proximal canopy (*CV_N_*) and intermediate canopy (*CV_M_*), which indicate the uniformity of the deposition distribution, were 8.56% and 12.33%, respectively.

## Data availability statement

The original contributions presented in the study are included in the article/Supplementary Material. Further inquiries can be directed to the corresponding author.

## Author contributions

The contribution of ShD is conceptualization, methodology, data curation, writing original draft and writing review and editing. The contribution of MO is methodology, writing review and editing, validation, and funding Support. The contribution of WD is data curation and methodology. The contributions of XY, XD, LJ, TZ, and SuD are methodology. The contribution of WJ is conceptualization, methodology, writing review and editing, validation, and funding Support. All authors contributed to the article and approved the submitted version.
